# Engineering Symbiotic Nitrogen Fixation for Agriculture: Predominant Role of Host Plants and Fine-Tuning Regulation

**DOI:** 10.3390/plants15081256

**Published:** 2026-04-19

**Authors:** Ting Wang, Fuxi Wang, Shuai Su, Luyang Yan, Zhiying Hao, Jinbo Xu, Huiru Han, Yan Wu, Dexiao Li, Senlei Zhang

**Affiliations:** 1 College of Grassland Agriculture, Northwest A&F University, Yangling 712100, China; tingting7336@163.com; 2 College of Agronomy, Northwest A&F University, Yangling 712100, China; wangfx0510@163.com (F.W.); shuaisu@163.com (S.S.); yanluyang323@163.com (L.Y.); ying88662026@163.com (Z.H.); xujinbo514@163.com (J.X.); hanhuiru0130@163.com (H.H.); wuyan0619@126.com (Y.W.); lidexiao@nwsuaf.edu.cn (D.L.)

**Keywords:** nodulation, legumes, bacteroids, engineering nitrogen fixation, sustainable agriculture

## Abstract

Symbiotic nitrogen fixation (SNF) can provide a sustainable and self-sufficient nitrogen (N) source for plants. Since its discovery, SNF has remained a central focus of both breeders and fundamental researchers. For decades, extending the utility of SNF to broader agricultural systems has been considered a promising strategy to reduce reliance on synthetic N fertilizers, thereby lowering production costs and mitigating environmental pollution caused by N overuse. This review summarizes recent advances in understanding the molecular and regulatory mechanisms governing SNF in legume plants and highlights emerging strategies to optimize and extend its application in agricultural systems. Particular emphasis is placed on approaches that aim to achieve dominant, fine-tuned, and controllable regulation of N fixation to support sustainable crop production.

## 1. Introduction

Although N is the most abundant element in the atmosphere, it is also one of the most essential and frequently limiting nutrients for plant growth. Nitrogen deficiency drives legume plants to establish symbiotic relationships with nitrogen (N)-fixing rhizobia residing in the rhizosphere [1]. Molecular communication mediated by plant-derived flavonoids and rhizobial lipochitooligosaccharides, known as Nod factors, bridges the two partners and initiates the formation of a highly specific and compatible symbiosis [2,3]. In response, host legumes develop specialized root organs termed root nodules to accommodate their rhizobial symbionts. Within root nodules, plants provide photosynthetically derived carbohydrates as an energy source and produce high levels of leghemoglobin, which creates a microaerobic environment that protects the oxygen-sensitive nitrogenase enzyme from inactivation [4]. This specialized environment enables the biological conversion of atmospheric N into plant-accessible ammonia. Unlike the fossil-fuel-intensive Haber–Bosch process, which operates at extreme temperatures and pressures, enzymatic N fixation occurs under mild physiological conditions. Characterized as a light-driven, near-zero-carbon process, SNF is an attractive alternative to synthetic N fertilizers for developing sustainable agriculture [5].

Engineering SNF in cereal crops, which account for the majority of global food production, has long been regarded as a major challenge and ultimate goal in plant biology [6,7]. Although the trade-off between fixed N and carbon assimilates can be beneficial, plant growth is largely constrained by photosynthetic capacity; therefore, engineered N fixation must be finely regulated by the plants. Over the past two decades, advances in cell biology, genetics, and molecular biology have substantially deepened our understanding of this complex symbiosis, bringing the long-standing vision of engineering SNF beyond legumes increasingly closer to realization [8,9].

## 2. Molecular Reprogramming and Reconstruction of Nitrogen Fixation

As a specialized organ evolved to support SNF, the root nodule provides a highly coordinated microenvironment that facilitates metabolite exchange, ensures adequate substrate supply, and maintains the physiological conditions required for efficient N fixation [10]. Consequently, transferring nodulation capacity to cereal crops represents an attractive—and potentially ultimate—strategy for conferring symbiotic N-fixing ability to non-legume plants. However, nodulation is an exceptionally complex developmental process that depends on a cascade of cellular signaling and cellular biological events. These processes are mediated by receptors and receptor-like kinases, downstream kinases, phytohormones, secondary messengers, transporters, transcription factors, and numerous downstream effector genes [8,11,12]. Reconstructing the complete nodulation signaling network in cereal crops would therefore require enormous effort and represents a long and challenging path toward the formation of functional N-fixing nodules.

Although root nodule symbiosis is phylogenetically restricted to specific lineages within the N-fixing clade, approximately 80% to 90% of terrestrial plants retain the ancient capacity to form symbiotic associations with arbuscular mycorrhizal fungi [10]. Deciphering the nodulation signaling pathway has revealed that several genes essential for SNF also function in mycorrhizal symbiosis, collectively constituting the so-called Common Symbiotic Signaling Pathway (CSSP). Both rhizobial Nod factors and fungal Myc factors—structurally related lipochitooligosaccharides—are perceived by LysM receptor kinases located on the plant root cell membrane. This perception leads to the activation of the plasma membrane-localized receptor-like kinase SYMRK and induces characteristic nuclear calcium spiking [13,14]. The calcium oscillations subsequently activate the calcium/calmodulin-dependent protein kinase CCaMK, which phosphorylates the transcription factor CYCLOPS. Once phosphorylated, CYCLOPS physically associates with DELLA proteins, which in turn interact with the GRAS family regulators NSP1–NSP2. Acting as a core transcriptional integration module downstream of the CSSP, this multiprotein complex responds to conserved calcium signaling and phytohormonal cues in both rhizobial and arbuscular mycorrhizal symbioses, thereby driving the expression of early symbiotic genes, cellular reprogramming, and the development of symbiotic structures [15,16,17,18,19].

Recent phylogenomic evidence suggests that the ancestral gene regulatory network of root nodule symbiosis (GRN-RNS) was established through the modular recruitment and functional rewiring of three conserved genetic programs: arbuscular mycorrhiza symbiosis, the nitrate response, and abiotic stress response pathways [10]. Many nodulation-related genes, including 419 RNS genes found to be recruited from the arbuscular mycorrhizal symbiosis pathway, are thought to have originated from the ancestral mycorrhizal symbiosis machinery through gene family expansion and neofunctionalization, as mycorrhizal symbiosis emerged hundreds of millions of years earlier than the evolution of legume–rhizobium interactions. Engineering nodulation in cereal crops by recruiting and repurposing mycorrhizal symbiosis genes may thus provide a more direct and feasible route toward establishing SNF in non-legume plants [20]. Mechanistic support for this concept comes from the identification of the formin protein SYFO2 as a key regulator of cytoskeleton-driven membrane invagination during symbiotic microbial accommodation. Importantly, SYFO2 also functions in the non-legume crop tomato, and its promoter remains responsive to the legume symbiosis regulator NODULE INCEPTION (NIN), suggesting that non-legume plants (e.g., cereal crops) may retain conserved genetic modules that could be exploited to engineer N-fixing symbioses [21]. In parallel with repurposing developmental pathways, components of plant immune signaling pathways can also be exploited, as substantial mechanistic parallels exist between immunity and symbiosis signaling [22]. For instance, recent studies demonstrated that although chitin elicitor receptor kinases (CERKs) are highly conserved immune receptors, they can be reprogrammed to perceive symbiotic signals by substituting only two amino acid residues within the “Symbiosis Determinant 1” (SD1) region, thereby enabling Nod factor recognition [23,24].

Beyond reconstructing nodulation capacity, direct transfer or utilization of the N-fixing machinery itself—either N-fixing bacteria (bacteroids) or the nitrogenase enzyme complex—represents an alternative and potentially more straightforward strategy. Nitrogenase is highly energy-demanding and extremely sensitive to oxygen, which is why root nodules provide an ideal niche for hosting N-fixing rhizobia. Recent advances have also indicated that mitochondria and chloroplasts may serve as suitable intracellular compartments for nitrogenase activity, as their highly efficient respiratory and photosynthetic metabolism can provide both low-oxygen conditions and sufficient energy supply. Over the past decade, several exciting engineering practices have demonstrated progress toward targeting nitrogenase components to these organelles, highlighting their potential as platforms for intracellular N fixation in plants [25,26,27,28,29,30,31,32].

## 3. Improving Nitrogen Fixation Efficiency Through Optimized Nodulation

Host plants tightly regulate nodule number to control the overall level of N fixation and balance its metabolic cost. Nodule number is influenced by multiple factors, among which infection efficiency of rhizobia plays a pivotal role, as it directly determines the initiation of nodule organogenesis. During evolution, legumes and rhizobia have developed finely tuned recognition mechanisms that enable the selective establishment of compatible symbiotic partnerships.

Legume roots secrete flavonoid and isoflavonoid compounds into the rhizosphere, which are perceived by rhizobial NodD proteins. The high specificity of flavonoid recognition is determined by polymorphisms in the three-dimensional structure of NodD, which can be dramatically influenced by a small number of amino acid residues. This structural sensitivity provides a promising avenue for protein engineering to broaden rhizobial host range, potentially extending compatibility even to non-legume plants [33].

Upon activation, NodD induces the synthesis and secretion of Nod factors, and successful symbiosis subsequently depends largely on the host plant cell-surface receptors that perceive and respond to these rhizobial signals. These receptors play a central role in determining host specificity and the range of symbionts. In addition to Nod factors, rhizobial infection is also modulated by the perception of other bacterial signals, including secreted effector proteins, microbe-associated molecular patterns, and cell-surface molecules [34,35,36]. For example, the rhizobial gene *rns1*, which encodes a type I-secreted protein, has been shown to restrict host range by interacting with the NS1a-NS1b receptor complex in *Medicago* [37]. In soybean, type III effectors (T3Es) secreted via the rhizobial type III secretion system (T3SS), such as Nodulation Outer Protein L (NopL), play critical roles in host range restriction and symbiosis establishment through interactions with host proteins including GmREM1a and GmNFR5 [38,39]. Engineering these interacting protein pairs has significant potential to extend symbiotic compatibility and improve infection efficiency.

Legumes have evolved a systemic mechanism, known as autoregulation of nodulation (AON), to limit nodule number. This long-distance signaling system integrates root-derived and shoot-derived signals to balance photosynthetic carbon allocation and nitrogen acquisition. Root-derived CLE (CLAVATA3/EMBRYO SURROUNDING REGION-RELATED) peptides, induced by rhizobial infection or external nitrate supply, are transported via the xylem to the shoot, where they act as negative regulators of nodulation. In contrast, the shoot-derived microRNA miR2111 promotes nodulation by repressing the accumulation of *TML1* and *TML2* (Too Much Love) transcripts in roots, which encode key suppressors of nodulation [40,41]. The integration of these signals is mediated by the nodulation autoregulation receptor kinases NARK (soybean), SUNN (*Medicago*), and HAR1 (*Lotus*), which fine-tune nodulation to optimize the cost–benefit balance. Recent CRISPR-Cas9-based mutant screens demonstrated that targeted modification of soybean RIC (rhizobium-induced CLE) peptides can increase nodule number without compromising plant growth or yield, providing a valuable proof of concept for nodulation optimization [42]. Acting downstream of the core regulator NIN, the miR172c module promotes nodulation by mediating the degradation of its target, the transcriptional repressor Nodule Number Control 1 (NNC1), thereby relieving the inhibition of early symbiotic signaling genes. Experimental evidence indicates that overexpression of miR172c significantly increases nodule density, underscoring the potential of manipulating this regulatory axis to optimize symbiotic N fixation efficiency in legumes [43].

In addition to the AON pathway, C-terminally encoded peptides (CEPs) regulate an independent systemic signaling pathway that promotes rhizobial infection and nodule formation. Although both CLE peptides and CEPs are induced by rhizobial infection and originate in the root, they exert antagonistic effects on nodulation. CEPs are transported to the shoot and perceived by the leucine-rich repeat receptor-like kinase COMPACT ROOT ARCHITECTURE 2 (CRA2). Activation of this pathway, particularly by CEP7, enhances nodulation by upregulating miR2111 expression in the shoot [44,45,46]. The dynamic balance between CLE and CEP signaling is integratively controlled by the NIN transcription factor, which plays a central role in maintaining optimal nodulation throughout plant development [47]. Notably, recent advances in CRISPR/Cas9 technologies have facilitated a comprehensive functional dissection of *NIN* across diverse legume models [48]. By deploying multiplex mutagenesis to overcome genetic redundancy in soybean and utilizing cell-type-specific promoters to bypass pleiotropic effects in *Medicago truncatula*, researchers have precisely elucidated NIN’s spatiotemporal roles without compromising basic plant development, thereby enabling a more refined optimization of the N fixation process.

## 4. Enhancement of Root Nodule Sustainability

Like other plant organs, root nodules have a finite lifespan, and their senescence may occur either as a part of normal development or in response to environmental cues. Nodule senescence marks the termination of SNF but also serves as an important nutrient recycling process, enabling the remobilization of N and carbon reserves stored within nodules. Nodule senescence can be induced by various environmental stresses, including shading, low temperature, drought, and salinity. In addition, excessive nitrate supply in the rhizosphere is a potent trigger of premature nodule senescence.

Nitrate application rapidly arrests nodule development and N-fixing activity. In *Lotus japonicus*, the NAC-type transcription factor LjNAC094 has been identified as a key positive regulator of nitrate-induced nodule senescence. Acting downstream of the nitrate-responsive transcription factors LjNLP1 and LjNLP4, LjNAC094 activates the expression of senescence-associated genes in response to nitrate signals [49]. Similarly, Soybean Nitrogen Associated NAPs (SNAPs), including SNAP1, SNAP2, SNAP3, and SNAP4, play critical roles in mediating nitrate-induced inhibition of nitrogenase activity and acceleration of nodule senescence [50]. These transcription factors reprogram the nodule transcriptome under N-rich conditions by directly regulating subnetworks of senescence-associated genes and transcriptional regulators, including NAC, WRKY, and ERF families.

A hallmark of both natural and stress-induced nodule senescence is a dramatic increase in proteolytic activity, which correlates with the strong induction of protease-encoding genes. Elevated expression of cysteine proteases has long been used as a molecular marker of nodule senescence and is accompanied by the downregulation and degradation of leghemoglobins. In soybean, the transcription factors GmNAC039 and GmNAC018 are key regulators of cysteine protease gene expression and play critical roles in controlling nodule senescence. Overexpression of *GmNAC039*, *GmNAC018*, or their downstream target genes encoding cysteine proteases (*GmCYPs*) markedly accelerates nodule senescence [51].

Root nodules supply large quantities of biologically fixed N to legume hosts. However, although N demand remains high throughout plant development, from germination to maturity, the functional lifespan of root nodules is often insufficient to fully meet this demand. This limitation is particularly pronounced in grain legumes such as soybean, where large-scale nodule senescence frequently occurs during flowering, leading to premature termination of SNF and substantial yield losses. Consequently, external N fertilizers are commonly applied to maintain high yields. While effective in the short term, this practice increases production costs and exacerbates nitrate-induced suppression of symbiotic N fixation, further shortening nodule lifespan. Current understanding of the developmental pathways that restrict nodule initiation (such as the AON pathway) has been largely established, which expands our perspective to the high-N responsiveness of mature nodules. Nevertheless, the precise molecular contours underlying how high-N stress breaks the homeostatic microenvironment of mature nodules, leading to the targeted inhibition of N fixation and the onset of programmed senescence via transcriptional rewiring, are still waiting to be uncovered [50,52]. Strategies aimed at delaying nodule senescence or alleviating nitrate-mediated nodule suppression through targeted modulation of key regulatory genes hold significant promise in the enhancement of N-fixing capacity not just in legumes, but also could support the design of more sustained and balanced N fixation systems in engineered crops. However, any such genetic manipulation must carefully account for the synergistic backlash from the accumulation of metabolic toxins, particularly reactive oxygen species (ROS) and nitric oxide (NO), alongside host immune defenses. As research advances to conceptualize symbiotic systems in engineered crops, addressing these same physiological constraints remains equally paramount. Successful extension of the N fixation period warrants a delicate orchestration of the host’s internal environment, ensuring that redox homeostasis is maintained and spurious immune responses are gently bypassed [8,51,53].

## 5. NCR Peptides as Emerging Targets for Engineering Symbiotic Nitrogen Fixation

As specialized niches for the reduction in atmospheric N, root nodules exhibit remarkable diversity in morphology and developmental programs across legume lineages. Two major nodule types are generally recognized: determinate and indeterminate nodules. Determinate nodules, commonly found in legumes such as *Lotus* and soybean, are typically spherical, lack a persistent meristematic region, and do not display a clear developmental gradient [54]. In contrast, indeterminate nodules, characteristic of legumes such as *Pisum* and *Medicago*, are elongated or branched and contain an active apical meristem. This meristem gives rise to a well-defined zonation along the proximal–distal axis, allowing the simultaneous presence of cells at distinct developmental stages.

In *Medicago*, *Pisum*, and *Aeschynomene* species, bacteroids undergo dramatic morphological transformations, adopting either elongated (E-morphotype) or spherical (S-morphotype) forms. These bacteroids are terminally differentiated, irreversibly losing the capacity to revert to free-living growth outside the nodule. In contrast, bacteroids in determinate nodules of *Lotus*, common bean, and soybean largely retain an undifferentiated rod-like morphology (U-morphotype).

Terminal bacteroid differentiation is mediated by a unique class of host-derived peptides known as Nodule-specific Cysteine-Rich (NCR) peptides. *NCR* genes are predominantly expressed in nodules of legumes belonging to the Inverted Repeat-Lacking Clade (IRLC), while homologs are largely absent from non-IRLC legumes such as *Lotus* and soybean. A notable exception is *Aeschynomene* spp. from the Dalbergioid clade, which expresses more than 80 *NCR*-like genes [55]. By simultaneously targeting of multiple bacterial cellular processes, NCR peptides suppress cell division, reduce proteome complexity, and increase membrane permeability, collectively driving rhizobia from a free-living to a highly specialized symbiotic state. These effects are thought to underlie the superior N-fixing capacity of terminally differentiated bacteroids relative to their undifferentiated counterparts [56,57,58].

The deployment of NCR peptides underscores the dominant regulatory role of the host plant in legume–rhizobium symbiosis and highlights the adaptive advantage of indeterminate nodules and terminal bacteroid differentiation. Recent studies employing near-isogenic rhizobial strains combined with integrated physiological, biochemical, and microscopic analyses have provided compelling evidence that indeterminate nodules—and the terminally differentiated bacteroids they host—exhibit higher N fixation efficiency [58]. *NCR* and *NCR*-like genes display highly dynamic temporal and spatial expression patterns within nodules [8,55,59,60,61,62], indicating functional specialization at different stages of nodule development. Mutation of *NCR169* (*dnf7*) results in nodules arrested at an early developmental stage: symbiotic cells become infected and contain elongated bacteroids, but these bacteroids lose viability due to activation of host defense responses, while the loss of the transcription factors MtAHLs, which severely disrupts *NCR* gene expression, leads to bacteroid death before differentiation occurs [63,64].

**Figure 1:**
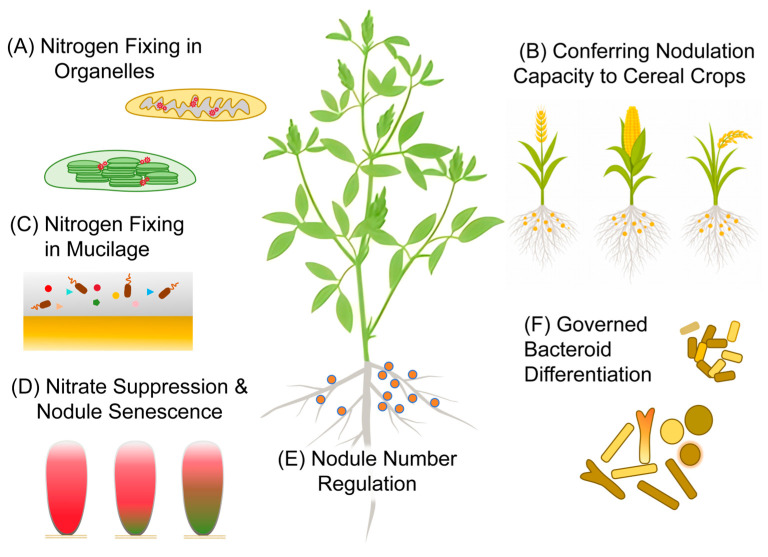
(**A**) Organelle-based nitrogen fixation: Non-legume nitrogen fixation may be achieved through synthetic biology approaches by expressing the nitrogenase complex in intracellular compartments such as mitochondria or chloroplasts, thereby converting them into “nitroplast”-like organelles; (**B**) Reconstruction of nodulation signaling pathways: Transfer of the complete nodulation signaling network holds the potential to confer nodulation capacity to cereal crops. (**C**) Association with free-living or engineered diazotrophs: Plant-secreted mucilage or xylem sap can provide both a micro-oxygen environment and carbohydrate supply to selected or engineered nitrogen-fixing bacteria. (**D**) Extension of nodule lifespan: Relief of nitrate-induced suppression and prolongation of nodule longevity through inhibition of nodule senescence can enhance the overall nitrogen contribution from SNF; (**E**) Optimized nodulation control: Fine-tuning nodule number and activity helps balance the cost–benefit trade-off of SNF, thereby maximizing crop yield under agricultural conditions. (**F**) Bacteroid differentiation-mediated enhancement: NCR and NCR-like peptides can be exploited to promote terminal differentiation of bacteroids, leading to increased nitrogen fixation efficiency.

Although the presence of NCR peptides is largely restricted to IRLC legumes—with limited exceptions such as *Aeschynomene* spp.—the transcription factors governing *NCR* expression are broadly conserved across legume species [64]. This conservation suggests that engineering strategies based on ectopic expression of *NCR* or *NCR*-like genes, driven by their native or nodule-specific promoters, may be feasible while preserving their tightly regulated spatial and temporal expression patterns. The extraordinary diversity of NCR peptides provides extensive opportunities to modulate bacteroid morphology and the extent of terminal differentiation; notably, *Medicago truncatula* alone is predicted to harbor more than 700 NCR family members. Beyond enhancing N fixation efficiency, ectopic *NCR* expression may also confer additional agronomic benefits, as the antimicrobial properties of these peptides could strengthen resistance to pathogens (Figure 1) [65,66,67].

## 6. Discussion and Future Perspectives

Over the past few decades, industrial N fixation via the Haber–Bosch process has played a crucial role in supporting global food security. However, fertilizer-dependent yield increases come at the cost of rising production expenses and severe environmental pollution caused by N runoff and leaching. As the global population is projected to reach 9 billion by 2050, the development of sustainable, environmentally friendly N sources has become increasingly urgent. Compared with the industrial production of N fertilizers, SNF in root nodules enables the conversion of atmospheric N under mild physiological conditions, using photosynthetically derived carbon as an energy source. This process represents a promising alternative to the excessive application of chemical N fertilizers in modern agriculture. SNF is highly efficient, supplying approximately 60–70% of the N required for legume growth. Since its discovery, transferring N-fixing capacity from legumes to non-legume crops, particularly cereals with high N demand, has been a long-standing aspiration in plant biology, driven by the need to sustain food production for a rapidly growing global population. During the past few decades, numerous strategies have been developed or are under development to utilize or maximize N-fixing ability. While in comparison, fine-tuning mechanisms that ensure N fixation to be proceeded under optimized level have received comparatively less attention and remain insufficiently understood.

Directly transferring N-fixing capacity to cereal crops appears to be a straightforward solution. With rapid advances in molecular biology and genetics, the core signaling pathways governing host–rhizobium recognition, infection, nodule organogenesis, and symbiosis maintenance have been largely elucidated. In principle, the stepwise ectopic expression of these components in cereal hosts may eventually be achievable. Due to their high respiratory activity, mitochondria and chloroplasts have been proposed as potential cellular compartments for hosting nitrogenase, as respiration not only reduces free oxygen to protect the enzyme but also supplies ATP for N reduction. Cereal crops, especially C4 species such as maize and sorghum, exhibit high photosynthetic efficiency and substantial N demand to sustain rapid growth and high grain yield, making them attractive candidates for engineered N fixation systems. Recent studies have suggested that mucilage secreted by cereal crops, particularly maize, as well as xylem sap, may be exploited to create micro-oxygen environments conducive to the growth of N-fixing bacteria [7,68,69]. Furthermore, a landmark discovery in 2024 revealed that the N-fixing cyanobacterium *Candidatus Atelocyanobacterium thalassa* (UCYN-A) functions as a novel N-fixing organelle, termed the “nitroplast”, in the unicellular alga *Braarudosphaera bigelowii*, opening new conceptual avenues for N fixation engineering [70].

Nevertheless, photosynthetic capacity remains a major constraint on yield improvement, particularly in C3 crops such as rice and wheat. N fixation is an energetically costly process, and excessive fixation may enhance vegetative growth at the expense of grain yield, which is largely composed of carbohydrates. Therefore, engineered N-fixing cereals must exert precise, dominant control over N fixation to maintain carbon–nitrogen balance and optimize yield. Such restriction could be implemented by recapitulating the legume AON pathway to construct a cereal-adapted negative feedback circuit that dynamically modulates N fixation according to real-time internal N supply and demand [42]. In parallel, the soybean GmNAS1–GmNAP1 sensory system can be used as a blueprint to identify cereal homologs, allowing fine-tuned allocation of phosphoenolpyruvate (PEP) between respiration and N fixation in response to cellular energy markers such as AMP [71]. Ultimately, the success of N-fixing cereals will hinge on the ability to install not only a functional “engine” for nitrogen fixation, but also a responsive robust “molecular brake” that ensures this engine operates at optimal capacity. Deciphering and reconstructing regulatory networks such as AON and nitrate-mediated suppression in a cereal context will therefore be as critical as, if not more than, the engineering of N-fixing ability itself.

In legumes, nodulation is under strict regulatory control. Host plants selectively recognize compatible symbionts through Nod factor receptors, type III secretion systems (T3SS), and recently identified regulators such as NCR peptides. Nodule number is controlled by the AON system, ensuring that only an optimal number of nodules develop. Additionally, the NLP/CLE/SUNN signaling pathway suppresses nodulation under high nitrate conditions, reflecting the host’s preference for external N sources when available. Host plants also coordinate nodulation with the acquisition of other mineral nutrients, including iron and phosphorus. For instance, nodulation is inhibited under iron limitation but stimulated when iron is sufficient through the action of the iron sensor BRUTUS A (BTSa) [72]. Similarly, miR399 has been proposed as a shoot-to-root systemic signal that suppresses nodulation under phosphate-deficient conditions [73]. Together, these mechanisms ensure host dominance in the symbiosis and maintain its overall benefit to plant growth and development. However, this regulatory architecture is inherently constrained by its temporal resolution. The formation of functional nodules typically requires 2–3 weeks, imposing a substantial lag between environmental fluctuation and physiological response. As a consequence, nodule number-based optimization strategy might fail to provide the precision or responsiveness required to optimize resource allocation for the host plants, especially under the contemporary climate scenarios.

In IRLC legumes, an additional regulatory layer enables finer control of SNF. NCR peptides, which are thought to have evolved from ancestral defensins, possess broad-spectrum antimicrobial activity and induce terminal differentiation of bacteroids within nodules. These differentiated bacteroids exhibit elongated, branched, or spherical morphologies and have been shown to fix N more efficiently than undifferentiated forms. Crucially, NCR peptides act directly at the level of the bacteroid—the smallest functional unit of nitrogen fixation—thereby enabling a degree of regulatory precision that is not achievable through other regulation mechanisms. Although the evolutionary advantage of maintaining hundreds of *NCR* genes remains unclear, accumulating evidence suggests that these diverse peptides may be exploited in engineered N-fixing systems to fine-tune bacteroid morphology, differentiation status, and ultimately carbon–nitrogen homeostasis. Emerging evidence supports the notion that distinct NCR peptides exert specialized and partially non-redundant functions, suggesting ultimate possibilities in the utilization strategy of NCRs as well as ultimate complexity in study them. The discovery that activators of *NCR* is widely presented among legumes provides an accessible entry point for functional characterization and cross-species deployment, which expands the feasibility of testing NCR function in heterologous systems. Looking forward, artificial intelligence-based tools, such as AlphaFold and related protein design platforms, offer new opportunities to model NCR structure–function relationships, predict peptide–target interactions, and rationally assemble optimized NCR combinations.

Overall, during the past two decades, advances in genetics and molecular biology have provided a detailed blueprint of root nodule development and symbiotic regulation. These breakthroughs have significantly accelerated efforts toward engineering N-fixing cereals. It appears that with continued progress, the long-standing goal of developing non-legume crops capable of efficient and controllable N fixation may become a reality in the foreseeable future. However, the rapid progress been made raises a fundamental concern among the authors that we might be approaching the capacity to install high performance N-fixing “engine” without yet possessing the means to control it, as the regulatory mechanisms required to govern this energetically intensive process remain comparatively underexplored. Existing regulatory frameworks, such as the AON network and nitrate-suppression pathways remain insufficiently characterized even within their native context, not mentioning to support the design of analogous feedback circuits in engineered plants. The emerging novel regulatory modules, such as NCR peptides, set paradigm for fine-tuning nitrogen fixation output at the cellular level, which could be extended to other potential N-fixing platforms—including engineered bacteroids, mitochondria, chloroplasts, or even the recently described nitroplast—remains largely unexplored. At the same time, advances in computational biology and protein design create opportunities to rationally engineer regulatory components, enabling the development of synthetic control modules tailored to specific physiological and environmental conditions. Adopting such approaches will be essential for moving beyond the mere installation of N-fixing capacity toward the creation of finely regulated, robust, and agronomically viable systems.

## Data Availability

The original contributions presented in the study are included in the article. Further inquiries can be directed to the corresponding author.
